# Best Practices for Supporting Occupational Therapy Students With Disabilities During Clinical Education: Results From a Three‐Round Delphi Study

**DOI:** 10.1155/oti/6675487

**Published:** 2026-09-01

**Authors:** Courtney Gohean, Katie McLaughlin, Aster Harrison

**Affiliations:** ^1^ Thomas Jefferson University, Philadelphia, Pennsylvania, USA, jefferson.edu; ^2^ Aix-Marseille Université, Marseille, France, univmed.fr

**Keywords:** accommodations, disability, fieldwork, occupational therapy

## Abstract

**Introduction:**

Disabled occupational therapy students face systemic barriers in clinical education. Although legal mandates require reasonable accommodations, implementation is often inconsistent, and few standardized guidelines exist for fieldwork education. This study is aimed at establishing best practices for supporting occupational therapy and occupational therapy assistant students with disabilities during fieldwork.

**Materials and Methods:**

A three‐round Delphi study was conducted with a panel of 30 experts, including disabled occupational therapy students, disabled occupational therapy practitioners, academic fieldwork coordinators (AFWCs), fieldwork educators (FWEs), disability services staff, and researchers. In Round 1, participants answered open‐ended questions about challenges, accommodations, and environmental supports. Responses were analyzed using conventional content analysis. In Rounds 2 and 3, participants rated the importance and feasibility of proposed practices using a 7‐point Likert scale. Practices with a median importance score of ≥ 6 and an interquartile range (IQR) < 2 were considered to have reached consensus.

**Results:**

Forty‐one best practices and 12 suggested available accommodations reached consensus for importance. The best practices were organized into six thematic categories: (1) facilitating communication, collaboration, and transparency; (2) advance planning; (3) normalizing and affirming accommodations; (4) education of key stakeholders; (5) individualized and flexible approaches; and (6) addressing structural barriers and institutional ableism.

**Discussion:**

Findings highlight the need for structured, inclusive approaches to disability accommodations in occupational therapy clinical education. Key recommendations include proactive communication, early planning, stakeholder education, and flexible implementation of supports. Many best practices emphasize cultural shifts towards disability affirmation, universal design for learning, and structural change—rather than relying solely on individualized accommodations.

## 1. Introduction

Disabled^1^ people are underrepresented among health professional students and workers in the United Kingdom, United States, Canada, and Australia [1]. Healthcare professional organizations have stated goals of increasing workforce diversity (e.g., [2, 3]), and reducing accessibility barriers for disabled practitioners could further these goals [4]. Additionally, disabled health profession students bring unique assets to their work due to their lived experiences of disability, including enhanced ability to empathize with patients [5], build rapport [6, 7], and advocate for disability‐affirming and inclusive services [7].

There is substantial evidence that health professionals demonstrate an implicit bias towards disabled people [8]. One study showed that while student′s explicit biases towards people with disability reduced over the course of their occupational therapy (OT) education, their implicit biases did not significantly change, and a majority of OT students demonstrated implicit preference for nondisabled people over disabled people at graduation [9]. Ableism refers to the systematic societal preference for able‐bodiedness over disability, which leads to exclusion and marginalization of disabled people [10]. A 2023 systematic review of 48 studies [11] explored ableism in the workplace for healthcare providers and disabled clinical students. The review reported widespread workplace ableism within healthcare occurring at individual levels (e.g., bullying) and institutional levels (e.g., environmental inaccessibility).

Past research has shown that academic and clinical professionals particularly struggle to support disabled health professional students during their clinical education [12–14]. Disabled health professional students encounter environments where they are excluded, undervalued, or perceived as less capable than their peers [15]. Beyond these interpersonal challenges, research highlights instances of discrimination [16–18] and a prevailing institutional discourse that frames disability as a deviation from the norm, effectively shifting the burden of accessibility onto the student [15, 17]. Some clinical instructors associate disabled students with concerns about patient safety ([19–23]) and an inability to meet practice standards ([19, 24, 12]). Clinical instructors often lack knowledge and understanding of disability accommodations [12–14], as well as the skills and confidence needed to support disabled students [14]. In a recent national study of Canadian fieldwork educators (FWEs) and academic coordinators, concerns were often framed around staff resources and the burden on supervisors, reflecting systemic barriers rather than students′ actual capabilities [19]. Furthermore, academic coordinators tended to hold more negative views than FWEs, sometimes viewing students needing accommodations as a potential risk to placement relationships [19].

Under the Americans with Disabilities Act and Section 504 of the Rehabilitation Act of 1973, postsecondary institutions in the United States are required to provide reasonable accommodations to qualifying students [25, 26]. This requirement extends to the school′s clinical education partners [27]. A clinical site must provide reasonable accommodations so long as they do not create an undue hardship [25, 28]. Accommodations are considered unreasonable if they impose an undue hardship on an organization, typically involving prohibitive financial costs or necessitating fundamental structural alterations [29]. International policies also protect disabled students′ rights to accommodations in higher education. The Convention on the Rights of People with Disabilities (CRPD) outlines access to higher education, including reasonable accommodations, as a key right of people with disabilities [30]. In the United Kingdom, protections against discrimination are established through the Equality Act 2010 [31], and more recently clarified in *University of Bristol v Abrahart* (2024), which emphasized that universities must actively collaborate with students to provide support. In Canada, the Human Rights Code [32] states that individuals with disabilities have the right to equal treatment in educational settings and are allowed accommodations up to the point of “undue hardship” [32]. However, research suggests that while higher education institutions and their partnering clinical sites are required to provide reasonable accommodations, their implementation in clinical settings remains inconsistent due to varied interpretations of undue hardship [13, 14, 18, 33, 34]. Due to ableism in healthcare education and workplaces, disclosure of disability is also a complicated decision for disabled health professional students [6, 12, 35, 36]. If a student perceives their instructors or environment as potentially unsupportive, they may choose to manage to the best of their ability without disclosing their disability, risking failing their clinical rotation instead of obtaining appropriate accommodations [12].

Although emerging literature, particularly in other health professional disciplines, has identified some useful practices in clinical accommodations [37], there still exists a dearth of clear models for fieldwork accommodations specific to OT [33]. Given the unique tasks involved in OT practice (e.g., activity analysis, environmental modification, and provision of adaptive equipment), the diverse practice settings, and the specific educational requirements, accommodation recommendations specifically designed for OT could be helpful to FWEs. Recognizing the need for discipline‐specific standards, this project engaged a diverse panel of stakeholders to formalize strategies tailored to the unique demands of OT clinical education. This educational research project used a three‐round Delphi process involving students and practitioners with disabilities, academic fieldwork coordinators (AFWCs), FWEs, and researchers to generate a consensus list of best practices for supporting occupational therapist (OT) and occupational therapy assistant (OTA) students with disabilities during Level II fieldwork.

## 2. Materials and Methods

### 2.1. Description of Delphi Process

The research team used a three‐round Delphi methodology to reach a consensus on best practices for working with disabled students on Level II OT or OTA fieldwork. The Delphi method invites experts to generate practice recommendations in an area where there is currently a dearth of evidence [38]. In the first phase of a Delphi study, purposively sampled experts share their opinions in a series of questionnaires [38, 39]. In the later rounds of the questionnaires, experts are provided with the medians of other experts′ responses and given the opportunity to adjust their original rankings in light of the group′s opinion. This methodology permits the remote contribution of experts who would be unable to participate in a consensus‐building process in person. This study followed in part the methodology utilized by Mirza et al. [40], including definitions of consensus. The DELPHISTAR checklist was used in preparing this article for publication [41].

### 2.2. Theoretical Framework

Disability studies scholars [42–44] advocate for greater integration of disability studies frameworks into health and rehabilitation education, promoting approaches that affirm the rights, autonomy, and dignity of disabled people. Aligned with this viewpoint, the social model of disability shifts the focus from the individual′s impairment to the broader societal and environmental factors that restrict participation [45]. Rather than viewing disability as an inherent problem located in an individual′s body, this model conceptualizes disability as a socially constructed phenomenon arising from inaccessibility and exclusion. According to this framework, people are “disabled” not by their bodies or diagnoses, but by systemic barriers such as discriminatory policies, inaccessible spaces, and negative societal attitudes. For instance, a student using a wheelchair may be perceived as disabled when faced with stairs to access a lab classroom; however, if the space included a ramp, the barrier—and thus the disabling experience—would be eliminated. The social model of disability was used as a guiding theoretical framework for this study.

### 2.3. Recruitment and Screening

The Delphi method requires an individualized definition of experts so that experts have the appropriate knowledge to contribute to the aim of the study. For this study, experts were individuals with deep knowledge of clinical accommodations in the OT field. Experts included the following:

• OT/OTA practitioners with a disability who graduated within the past 5 years and had a disability during their Level II fieldwork^2^.
• OT/OTA students with a disability who have completed at least one Level II fieldwork placement.
• OT/OTA FWEs (clinicians) who had at least one student with documented disability accommodations for Level II fieldwork in the past 5 years.
• AFWCs with at least 3 years of experience who have supported at least two students with documented disability accommodations for Level II fieldwork.
• Researchers who have published on the topic of OT practitioners/students with disabilities and/or clinical accommodations in the past 10 years.
• Staff members at a university disability services office who had supported at least two OT or OTA students with documented disability accommodations in preparing, communicating, and/or navigating disability accommodations for fieldwork.

Given that the role titles for these people vary significantly across institutions and countries, the authors′ definitions of the key people involved in supporting students with disabilities in OT clinical education are provided in Table 1 to assist the reader in adapting study findings to their local context.

**Table 1:** Definitions of key people in the accommodation process for OT/OTA students with disabilities.

Definitions of key people	
Party name	Definition
Academic fieldwork coordinator (AFWC)	A member of the university faculty or teaching team in the OT or OTA program, who coordinates clinical placements for students.
University disability resource professional (DRP)	A university staff member who is responsible for coordinating disability accommodations for students with disabilities. They are usually responsible for students in many different academic programs at the university (not exclusively OT/OTA). The university departments where these professionals work are sometimes called disability resources centers, accessibility offices, disabled student resource centers, and so on.
Fieldwork educator	A licensed OT or OTA clinician who supervises a student OT/OTA in the clinic during the fieldwork. In other fields, this person is often called a “clinical instructor” (CI).
Profession	The occupational therapy profession as a whole, including groups and communities of practitioners, and especially leadership organizations. Includes professional associations including American Occupational Therapy Association (AOTA) or the World Federation of Occupational Therapy (WFOT) as well as educational entities such as the National Board for Certification in Occupational Therapy (NBCOT) in the United States, or the European Network of Occupational Therapy in Higher Education (ENOTHE).
School (university, academic program)	The university and/or academic program where the student is enrolled and which will issue their degree.
Site	The clinical site where the FWE works and where the student will be placed. Can be a hospital, clinic, community organization, or any other site where an OT or OTA might work.
Student	The OT/OTA student

Recruitment followed a purposeful sampling approach, including contacting researchers who have published in this subject area, emailing recruitment letters to OT program directors and AFWCs located in the United States, and sharing informational virtual flyers on OT/OTA Facebook groups. Potential experts completed a screening survey. Eligible experts were then contacted via email and invited to participate in the Delphi study. Demographic information of selected experts was collected through the screening survey, downloaded into Microsoft Excel, and analyzed descriptively. All questionnaires were delivered online via Qualtrics [46].

A recent meta‐research study reports that general guidance for Delphi study sample sizes recommends a sample of 15–30 individuals for heterogeneous panels [47]. Other classic methodological resources suggest a sample size of 10–18 experts [48]. Attrition is to be expected in multiround Delphi studies [47]. To have enough experts for each ranking, we set a target minimum sample size of 20 per round.

### 2.4. Participant Consent and Payment

This study received ethics approval and oversight from the Institutional Review Board of Thomas Jefferson University (iRISID‐2023‐2175). All participants provided written consent online via Qualtrics prior to completing the screening questionnaire. Participants were not compensated for the screening questionnaire. Selected eligible participants were compensated $30 via online Visa gift card after completion of each of the three Delphi rounds, for a total possible compensation of $90. Participants were free to withdraw from the study at any time.

### 2.5. Round One

The first Delphi round was conducted in April 2024. Experts were asked to complete four open‐ended questions about challenges with fieldwork accommodations, best practices for accommodations, and environmental supports and barriers. The first‐round survey contained primarily open‐ended questions, developed from review of scientific literature about healthcare professions students′ clinical training. The format of all surveys was inspired by the design of Mirza et al.′s [40] Delphi study. The Delphi questionnaires are available in the Supporting Information associated with this article. The open‐ended responses for Round 1 were qualitatively coded using conventional content analysis [49] by two coders (first and last authors). Qualitative responses for each round were compiled and analyzed anonymously, taking the experts as a group and looking across responses for similarities and differences (horizontal analysis), rather than analyzing vertically by participant or demographic groups. The researchers thoroughly read responses and independently noted initial impressions (memos) and initial codes. Then, two rounds of coding were completed and discussed, identifying key concepts and refine categories and subcategories. Disagreements between the two coders were resolved via discussion and consensus.

### 2.6. Round Two

The second Delphi round was conducted between May 2024 and June 2024. Experts were asked to rate the practices identified through content analysis of Round 1 responses on a 7‐point Likert scale for importance and feasibility (from 1—*strongly disagree* to 7—*strongly agree*). Experts were also provided the option to suggest edits or additions to each practice. Results were downloaded to Microsoft Excel to calculate the interquartile range (IQR) to determine consensus. To reach consensus, practices needed a median score of 6 (agree) or higher for importance and an IQR less than 2 used by Mirza et al. [40]. Feasibility medians were also calculated, but the feasibility score was not used to determine consensus. Qualitative comments on open‐ended questions were also analyzed with directed content analysis by two coders (first and last authors). These qualitative comments permitted revision and addition of several items to the Round 3 survey.

### 2.7. Round Three

The third Delphi round was conducted between June 2024 and July 2024. Experts were asked to rerank items that did not reach consensus in Round 2. During this process, they were shown the median score of the other experts for each item to decide if they wanted to change their Round 2 score in light of other experts′ views. This participatory consensus building strategy is a hallmark of the Delphi method [38]. The medians and IQRs were calculated for Round 3 responses, and practices with a median score of 6 (agree) or higher and an IQR less than 2 were considered to have reached consensus. Qualitative responses were again analyzed with content analysis by two coders to inform final organization into themes. Participants′ identities remained confidential throughout the research, such that items were anonymized, and participants saw only the median rating given by other experts.

### 2.8. Organization Into Themes

Once the best practices were finalized and validated by the experts, the three authors used directed content analysis [49] to organize best practices into themes and identify relevant stakeholders. These themes are a post hoc organizational tool intended to aid understanding and use of the best practices. Practices were first organized by “key people” (the person or people responsible for implementing the practice), then went through several iterations of conceptual categorization before settling into the final categorization presented in this article. A total of six themes were created for the *best practices.* Note that many best practices could have been categorized into multiple themes due to the complexity and interplay among themes. The three researchers worked together through discussion to reach consensus on the most relevant theme for each item. The *suggested available accommodations* remained in a separate category and were not reorganized at this stage.

### 2.9. Research Team and Reflexivity

All members on the research team are occupational therapists licensed in the United States. One author is a current OT faculty member in the United States, and one author was formerly OT faculty in the United States and now lives and works in France. The authors′ educational backgrounds include: one PhD in disability studies, one EdD with a focus on accommodations in fieldwork, two clinical doctorates in OT, and one clinical master′s degree in OT. All authors are white, two are women and one is a man. Two authors are heterosexual, and one is queer. Two authors identify as disabled, and one author identifies as nondisabled.

The authors drew upon their experiences in academia and clinical practice, as well as lived experience of disability, to make sense of the data and sort the findings into usable practices. The researchers reflected together on similarities and differences between their professional and personal experiences and the research findings. The use of multiple coders helped ensure that all practices were grounded in the collected data.

### 2.10. Use of AI

ChatGPT was trialed to organize the final 41 best practices into thematic categories. The final practices were entered into ChatGPT with a prompt to organize them into themes. The AI themes were not fully logically coherent and were therefore not adopted for the final themes, but this did serve as a part of the brainstorming and discussion process. ChatGPT was occasionally used for checking reference style.

## 3. Results

### 3.1. Expert Characteristics

Table 2 reports the demographic characteristics of all participants in Round 1 of the Delphi process who provided qualitative responses that were coded into the items that would be ranked in the following rounds. A majority of the participants were located in the northeast of the United States. There were eight participants who resided in the midwest, five participants from the east coast, and one individual who lived on the west coast. Of the 30 participants who completed Round 1 of the Delphi survey, 26 participants completed Round 2 of the survey, and 23 participants completed all three rounds of the survey. The individuals who dropped out during Round 2 were all practitioners. During Round 3, dropouts were three AFWC and one FWE. All dropouts were women, six individuals were white, and one individual was black. Three dropouts were bisexual/pansexual and the remaining four were heterosexual.

**Table 2:** Demographic characteristics of participants.

Total experts	*N* = 30
Roles^a^	
Academic fieldwork coordinator (AFWC)	9
Practitioners with disabilities (six OTs and three OTA)	9
Students	5
Fieldwork educators (FWE)	4
Disability services staff members	2
Researchers	2
Years of experience (*n* = 20)^b^	
< 1	3
1–5	14
6–10	3
> 11	3
Gender	
Woman	25
Man	4
Nonbinary	1
Sexuality	
Heterosexual	21
Bisexual/pansexual	5
Gay	2
Lesbian	1
Prefer not to answer	1
Age range	22–65 (mean = 35.63)
Race	
White	25
Black/African American	2
Mixed race	2
Asian American/Asian	1
Disability status	
Disabled	20
Nondisabled	10
Disability type^a^	
Psychological	12
Cognitive or learning disability	10
Chronic illness	5
Sensory	3
Short‐term physical disability	1
Mobility and/or physical	3
Disability visibility (*n* = 20 people with disabilities)	
Rarely/never	10
Sometimes	8
Always or almost always	2
Settings^a^	
Skilled nursing facility	9
Acute care or inpatient rehabilitation^c^	8
Pediatric outpatient	7
Adult outpatient	4
Mental health	4
School	4
Home health	3
Academia	2
Seating and positioning via telehealth	2
Assistive technology	1

^a^Experts were able to select multiple responses.

^b^Years of experience do not include information from researchers, disability staff, and students.

^c^Due to unclear language in the open‐ended responses about hospital‐based OT settings, the two categories were combined.

### 3.2. Round One Results

Content analysis of the qualitative responses provided on the Round 1 survey yielded 52 potential practices for supporting OT/OTA students with disabilities on Level II fieldwork. Forty of these practices were categorized by the researchers as “best practices” which could be approaches adopted by key people or systems to support most or all students with disabilities. In addition to these 41 *best practices*, experts also provided several *suggested available accommodations*. *Suggested available accommodations* were individual accommodations that a student might receive on fieldwork, but which would not be applicable to all students. Many participants very strongly advocated for these potential accommodations, so the researchers elected to have the experts vote on their importance and feasibility at Round 2. Twelve of these suggested accommodations were sufficiently salient during content analysis to make a final list for Round 2. However, since they were not general *best practices*, the questionnaire prompted participants to think of these 12 statements as menu items of accommodations that might possibly be made available to an eligible student, rather than general best practices for all students. See Supporting Information for a copy of the questionnaire (available here)

### 3.3. Round Two Results

Forty‐seven of the 52 items generated from Round 1 reached consensus for importance at Round 2 and were adopted as part of the final list. Five items did not meet consensus for importance. These were the following:

• Standardized accommodation process at school and sites (consistent process/timeline across students, even if individual accommodations are tailored to each student).
• Student and AFWC complete site visit before fieldwork to assess accessibility.
• Support for and recognition of the labor and time required of students to navigate accommodations (e.g., time for the accommodations process from start to finish, how Level II fieldwork accommodations like extra time may cut into other life activities for students).
• Reduced productivity expectations for FWEs while supporting students.
• Reduced caseload or slower increase in caseload

The final item “Reduced caseload or slower increase in caseload” was a *suggested available accommodation*, whereas the other four were general *best practices.*

### 3.4. Round Three Results

Following the Delphi process, the five items that did not meet consensus at Round 2 were included for reranking on the Round 3 questionnaire. “Reduced caseload or slower increase in caseload” was separated into two different practices, one for “reduced caseload” and one for “slower increase in caseload,” in response to participant comments at Round 2. Slower increase in caseload reached consensus for importance in Round 3, whereas reduced caseload did not.

Reworded versions of the other four practices (standardized process, site visit, support and recognition for labor, and reduced productivity expectations for FWEs) all reached consensus for importance at Round 3. The final wording of these practices is visible in the results tables.

In analyzing the Round 2 results, the researchers noticed that the qualitative comments indicated that participants were sometimes conflating their importance and feasibility rankings. For example, comments on the “standardized process” item included: “I know for my school, this process wouldn′t be feasible” and “The only reason I think this is a potentially challenging one is that every school and site will handle things a little bit differently”. In Round 3 on the item for importance ranking, participants received a reminder that read, “Please remember that when you are scoring items for importance, you do not need to consider feasibility.”

At Round 3, the researchers also added two new items for ranking, based upon the qualitative comments received during Round 2. These were as follows: “Training for university student disability services staff (disability resource professionals [DRPs]) providing background about OT/OTA, clinical rotations/fieldwork, and clinical accommodations to better prepare DRPs to support OT/OTA students with disabilities.” (suggested *best practice*) and “Dedicated/scheduled breaks in the student′s daily fieldwork schedule, e.g., for lunch, using the bathroom, taking medication, etc.” (*suggested available accommodation*). Both of these new items reached consensus for importance at Round 3.

This led to a final list of 53 items (41 *best practices* and 12 *suggested available accommodations)* that reached consensus for importance.

### 3.5. Final Results by Theme

The final 41 *best practices* were organized into six themes: (1) facilitating communication, collaboration and transparency; (2) advance planning; (3) normalizing and affirming disability accommodations, taking a disability rights approach; (4) education of all key people; (5) individualized and flexible approach to accommodation; and (6) addressing structural barriers and institutional ableism. Each of the best practices is presented in thematic tables with their median importance and feasibility scores from the round when they reached consensus, as well as the key people who have a primary role in implementing the practice. The 12 *suggested available accommodations* are also presented in their own thematic category.

#### 3.5.1. Facilitating Communication, Collaboration, and Transparency

Ten of the best practices, described in Table 3, focus on clear communication, collaboration, and transparency between the parties involved in supporting OT and OTA students on fieldwork, including: AFWCs, university DRPs, FWEs, fieldwork site, students, and universities/academic programs. These key people are defined in Table 1. The practices associated with this theme highlight the importance of keeping the student informed at all steps of the accommodation process, communicating early and clearly about expectations and problems on fieldwork, and open collaboration. The accommodation process is complex and involves multiple stakeholders. The experts were clear that open communication between all parties is crucial for success.

**Table 3:** Best practices for Theme 1: facilitating communication, collaboration, and transparency.

Facilitating communication, collaboration, and transparency			
Best practice	Median importance score (out of 7 possible)	Median feasibility score (out of 7 possible)	Key people
Transparency about the accommodations process—the school and fieldwork site keeping the student informed and included directly in conversations about accommodations.	7	7	AFWC, DRP, FWE, site
Fieldwork educator provides students with positive feedback (not exclusively critical feedback).	7	7	FWE
Student engages in self‐advocacy for accommodations.	7	7	Student
Fieldwork educator sets and communicates clear expectations for the student about the skills and progress they are meant to show on fieldwork.	7	7	FWE
Collaboration between academic fieldwork coordinator, fieldwork educator, student, and school′s disability services office before the fieldwork (involving all parties working together and communicating openly).	7	6	AFWC, DRP, FWE, site, student
Regular check‐ins between student, site/fieldwork educator, and academic fieldwork coordinator while on fieldwork to identify and address problems early (e.g., if student is not meeting expectations).	7	6	AFWC, FWE, site, student
The academic fieldwork coordinator and school′s disability services office individualize accommodations for each student.	7	6	AFWC, DRP, student
Academic fieldwork coordinator provide templates and communication aids for conversations between fieldwork educator and student about accommodations, learning styles, and expectations.	6	6	AFWC, FWE
Regular check‐ins or feedback process for the student to provide feedback to the academic fieldwork coordinator and the fieldwork educator about whether accommodations are effective and being provided as planned—without fear of retaliation.	6	6	AFWC, FWE, student
Fieldwork educator provides student feedback in multiple formats (e.g., written, verbal, and video analysis).	6	6	FWE

#### 3.5.2. Advance Planning

Preparing accommodations in advance of the fieldwork as much as possible supports student success. Five of the best practices, shown in Table 4, spoke to the importance of advance planning. Experts recommended that this planning start at the beginning of the academic program, by informing students about their rights to accommodations early in the curriculum and helping them brainstorm what accommodations they might need during clinical fieldwork while they are still completing their didactic coursework. Students′ experiences in the classroom, and particularly in lab or hands‐on activities, can help predict what they will need on fieldwork. Once a fieldwork site has been selected, if the student is willing to disclose their disability in advance, it can be helpful to discuss accommodations with the site and the FWE before the fieldwork starts. A site visit, whether virtual or in‐person, can also help the student, site, FWE, and AFWC plan for success—for example identifying accessibility barriers in advance, finding calm spaces that work for students with an accommodation for quiet documentation space, and mentally preparing the student for their first day.

**Table 4:** Best practices for Theme 2: advance planning.

Advance planning			
Best practice	Median importance score (out of 7 possible)	Median feasibility score (out of 7 possible)	Key people
Academic fieldwork coordinator, faculty, and school′s disability services office educate students about accommodations early in the curriculum (e.g., first semester, with reminders each semester).	7	7	AFWC, DRP, school
Advance planning of accommodations with fieldwork educator and student well before fieldwork begins (involves early student disclosure of accommodation needs).	7	6	AFWC, FWE, student
Faculty, academic fieldwork coordinator, and school′s disability services office help students brainstorm accommodations and understand the differences between classroom and clinical accommodations (ease the transition from classroom to workplace).	7	6	AFWC, DRP, school, student
Schools and sites have a developed, standard, step‐by‐step process for how they implement accommodations. For example, they would have a readily available list of the steps of the process, accommodations commonly used, key people to involve, and other information necessary to the process. Each school and site′s process could be different, but within their organization they would have a “standard of practice” or workflow/checklist established^a^.	6	5	School, site
Student and academic fieldwork coordinator complete site visit (in person or virtual) before fieldwork to assess accessibility^a^.	6	5	AFWC, site, student

^a^Reached consensus for importance at Round 3.

#### 3.5.3. Normalizing and Affirming Disability Accommodations, Taking a Disability Rights Approach

Eight of the final best practices, shown in Table 5, emphasized the importance of a disability rights approach to the accommodation process, which normalizes and affirms the inclusion of disabled practitioners in the OT/OTA workforce. Experts agreed that the attitudes of the professionals involved in the accommodation process matter and that approaching accommodations as natural, expected, and a basic student right supports student success on fieldwork. Attitudes regarding disability in the wider profession are also a crucial area for intervention—experts agreed that the profession and national membership organizations could support students with disabilities by taking public stances that affirm the value of disabled practitioners for the workforce and the people OT practitioners serve. Educational institutions and organizations can also better support students with disabilities by defining entry‐level competences, which students must demonstrate at the end of fieldwork in a disability‐affirming way. For example, acknowledging that not all practitioners need to work full‐time, and that collaborative approaches (e.g., directing transfers rather than physically completing them) are acceptable and even valuable.

**Table 5:** Best practices for Theme 3: normalizing and affirming disability accommodations, taking a disability rights approach.

Normalizing and affirming disability accommodations, taking a disability rights approach			
Best practice	Median importance score (out of 7 possible)	Median feasibility score (out of 7 possible)	Key people
Academic fieldwork coordinator advocates for and supports the student in accommodations process.	7	7	AFWC
Fieldwork educator, academic fieldwork coordinator, and school disability services office approach accommodations as an access and equity issue (a necessity and not a favor).	7	7	AFWC, FWE, DRP
Fieldwork educator, academic fieldwork coordinator, and school′s disability services office conceptualize entry‐level practice expectations in a disability‐inclusive way (e.g., recognize that not all OT practitioners need to work full‐time and different settings have different demands).	7	7	AFWC, FWE, DRP
Fieldwork educator, academic fieldwork coordinator, and school′s disability services office normalize the requesting of accommodations—approaching this as something natural and acceptable (not stigmatized).	7	6	AFWC, FWE, DRP
OT professionals and [national professional organizations] acknowledging and valuing students and practitioners with disabilities.	7	6	Profession
Supportive/affirming attitude of fieldwork educator, academic fieldwork coordinator, fieldwork site, school, and student disability services regarding disability and accommodations.	7	6	AFWC, FWE, DRP, school, site
Fieldwork educator and academic fieldwork coordinator create a fieldwork environment where students feel safe to make mistakes.	7	6	AFWC, FWE
Fieldwork educator, academic fieldwork coordinator, and school′s disability services office demonstrate an awareness of and avoidance of disability stigma and bias.	7	6	AFWC, FWE, DRP

#### 3.5.4. Education of All Key People

Six practices, detailed in Table 6, focused on the importance of educating all of the people involved in the student accommodation process. For AFWCs, experts explained that AFWCs need to be knowledgeable about accommodations, disability rights, and the relative accessibility of different fieldwork sites, and that they have a role in educating FWEs and fieldwork sites about the accommodation process. FWEs could benefit from resources about disability accommodations, whether these are provided by their work site, the school, the AFWC, or the profession. University disability resource professionals can benefit from information about OT and OTA clinical roles in order to better assist students in setting up reasonable accommodations. Finally, students themselves can benefit from mentorship from practitioners with disabilities to learn to better navigate the profession.

**Table 6:** Best practices for Theme 4: education of all key people.

Education of all key people			
Best practice	Median importance score (out of 7 possible)	Median feasibility score (out of 7 possible)	Key people
Academic fieldwork coordinator should be knowledgeable about disability, accommodations, and disability policies.	7	7	AFWC
Academic fieldwork coordinator educates sites and fieldwork educators on accommodations process and accessibility.	7	6	AFWC, FWE, site
Academic fieldwork coordinator demonstrates strong understanding of how/which sites are accommodating.	7	6	AFWC
Education/training and/or resources for fieldwork educators on disability, accommodations, and disability policies.	7	5	AFWC, FWE, profession, school, site
Training for university student disability services staff (disability resource professionals [DRPs]) providing background about OT/OTA, clinical rotations/fieldwork and clinical accommodations to better prepare DRPs to support OT/OTA students with disabilities^a^.	6	6	DRPs, school
Students receiving mentorship from practitioners with disabilities.	6	5	Profession, school, student

^a^New practice at Round 3, proposed from Round 2 comments. Reached consensus at Round 3.

#### 3.5.5. Individualized and Flexible Approach to Accommodations

Four best practices, detailed in Table 7, emphasize that while there are some general approaches and standards that can be encouraged, disability accommodations remain highly personal, and the process will often need to be individualized for each student. AFWCs are advised to do their best to matchmake between site accessibility and student needs. FWEs can support students by adapting their feedback and teaching style to fit student preferences—which involves openly communicating with students about their needs and preferences. While advance planning is helpful when possible, all parties involved in the accommodation process also need to support the students′ decisions about self‐disclosure of disability—including if this means they disclose later in the process, disclose to some sites and not others, or decide to not disclose at all. For students with less‐visible disabilities, they are often weighing the risks of discrimination and stigma with the benefits of accommodations that could come from disclosure. Professionals can help students think through these pros and cons, but part of a disability rights approach is supporting the student′s right to self‐determination in matters of disclosure and accommodation. Finally, experts agreed it is important that all parties recognize that disability is fluid, and that students′ needs—and therefore their possible accommodations—might change during the fieldwork. Student accommodations can change during fieldwork either when students develop new impairments, or when they realize through experiential learning during the fieldwork that they need other accommodations they did not anticipate. While these changes can be challenging, this is a crucial part of the clinical learning process for students with disabilities.

**Table 7:** Best practices for Theme 5: individualized and flexible approach to accommodations.

Individualized and flexible approach to accommodations			
Best practice	Median importance score (out of 7 possible)	Median feasibility score (out of 7 possible)	Key people
Fieldwork educator, academic fieldwork coordinator, and school′s disability services office support the student′s decision about whether or how to disclose disability and/or accommodation needs (e.g., help student weigh pros and cons of early disclosure, understand risks of disclosure, and respect student privacy).	7	7	AFWC, DRP, FWE
Academic fieldwork coordinator, fieldwork educator, and student recognize that accommodations and its process are fluid; students′ needs may change before or during the Level II fieldwork.	7	6	AFWC, FWE, student
Fieldwork educator teaches and offers feedback with student′s learning style in mind.	7	6	FWE
Academic fieldwork coordinator matches student accommodation needs with the site/setting (e.g., thoughtfully considering pace, productivity expectations, site resources, site accessibility, and proximity to student′s home/medical appointments).	7	5	AFWC

#### 3.5.6. Addressing Structural Barriers and Institutional Ableism

The eight best practices associated with the final theme, *addressing structural barriers and institutional ableism*, are described in Table 8. These best practices refer to changing university, site, or profession‐wide standards to set norms that are more universally designed to promote inclusion of people of different ability statuses.

**Table 8:** Best practices for Theme 6: addressing structural barriers and institutional ableism.

Addressing structural barriers and institutional ableism			
Best practice	Median importance score (out of 7 possible)	Median feasibility score (out of 7 possible)	Key people
University′s technical standards should outline the performance outcome rather than the student′s physical or mental abilities (i.e., what the student needs to do rather than how they need to do it) and specify that they can be met with or without accommodation. For example, specifying students are required to “communicate effectively” rather than saying they are required to “communicate verbally,” leaving open the possibility for students who communicate with sign language or interpreters to still meet the technical standard.	7	6	School
Inclusion of content about disability accommodations and access in [national OT educational] standards.	7	6	Profession
Site environments with elements of universal design—supportive features in the social and physical environment that support all people even in the absence of accommodations. For example, elevators or a quiet space available to all people.	7	5	Profession, site
Sites take a strong team/collaborative approach to workload and supervision rather than a competitive and productivity‐focused approach.	7	5	Profession, site
Fieldwork educators have reduced productivity expectations when supporting any student (not just students with disabilities) during Level II fieldwork. This could be through reducing caseload or productivity expectations, or through reconceptualizing teaching/mentoring as part of productivity^a^.	7	4	Profession, site
OT practitioners and [national professional organizations] advocating for/facilitating a culture shift in the profession to address ableism.	6	6	Profession
Recognizing that students with certain accommodations may end up working many more hours outside of fieldwork to do documentation, treatment planning, and so on with an extra time accommodation. Additionally, before fieldwork, students with accommodations may spend many extra hours outside of schoolwork to pursue accommodations. Also, recognizing the emotional weight of self‐advocacy labor^a^.	6	5	AFWC, DRP, FWE, profession, school, site
Reduced emphasis on productivity in the profession as a whole.	6	3	Profession, site

^a^Reached consensus for importance at Round 3.

One example of structural change at the university level is the drafting of more inclusive university technical standards. OT programs in the United States outline “technical standards,” which describe the essential abilities students must be able to demonstrate, with or without reasonable accommodation, to progress through the academic program and graduate. Similarly, Canadian OT programs have outlined “essential skills and attributes” that students must demonstrate to successfully complete training. Although policies in both the United States and Canada specify that students can use reasonable accommodations to meet these standards, their language often emphasizes physical health, sensory abilities (e.g., hearing, vision, and touch), and emotional resilience as baseline requirements, positioning the “healthy” student as the default [50]. Such phrasing reflects a medicalized or deficit perspective of disability, which may unintentionally exclude students with chronic illness, mental health conditions, or physical or sensory differences. Revising these standards with more inclusive language could help ensure that competence is defined in ways that recognize diverse ways of performing skills, thereby reducing structural ableism within entry requirements. For example, experts suggested writing that students are required to “communicate effectively” rather than saying they are required to “communicate verbally,” leaving open the possibility for students who communicate with sign language or interpreters to meet the standard nonverbally. Drafting technical standards with inclusive language is a form of institutional universal design, potentially allowing disabled students to meet standards even without formal accommodations.

Experts also suggested that curricular content about disability accommodations and accessibility be specifically included in educational standards for OT programs. They also wanted to see public statements of support and advocacy for disability rights from national professional organizations. Experts also agreed on the importance of fieldwork sites that already have environmental accessibility features in place before welcoming a disabled student—especially sites that contain elements of universal design. These best practices speak to the need for a broader cultural shift to view disability as a form of desired diversity in the profession.

A final subset of these best practices pertained to structural barriers associated with productivity standards in US OT practice. In many OT positions in the United States, supervisors calculate the percentage of time a therapist spends in direct patient care compared with the time they spend documenting their session in the electronic medical record. These data are used to calculate a productivity percentage, so for example if a practitioner saw patients for 7 h and spent 1 h on other tasks (such as team meetings, documentation, educating a student, looking for a resource for a client or colleague, and asking advice of a colleague), their productivity would be 87.5%. This can be very challenging to meet given the high expectations for documentation (e.g., expecting that independence levels of ADLs are documented every day in the hospital) in the United States. Experts stated that productivity expectations could be a barrier to good teaching practices for clinician educators, whose teaching time usually counts as a “nonbillable service” in their productivity calculations—so they can be penalized for taking extra time to teach a student. High productivity expectations are also often a barrier for students with disabilities, who in some cases may require more time for documentation, especially given the lack of accessibility features in many electronic medical software programs and OT workspaces. Experts agreed that reconceptualizing the profession′s approach to productivity would be beneficial to students with disabilities.

#### 3.5.7. Suggested Available Accommodations

The *suggested available accommodations*, shown in Table 9, were not conceptualized by or presented to the Delphi experts as a comprehensive list of the accommodations that OT/OTA students might need. Rather, participants in this study wanted to mention these accommodations because they are often overlooked, or because they were particularly important in their personal experience. These suggested accommodations are included here as they might be helpful as a menu of options to aid students and their supporters in brainstorming their needs and the possible accommodations for fieldwork, but they are not general principles that should be applied to all students with disabilities.

**Table 9:** Suggested available accommodations.

Suggested available accommodations		
Best practice	Median importance score (out of 7 possible)	Median feasibility score (out of 7 possible)
Wheelchair/physical accessibility (ADA compliance) at sites.	7	6
Use of assistive technology for note taking and memory (e.g., audio recording, talk‐to‐text assistive technology, and note‐taking aids).	7	6
Fieldwork site in close proximity to student′s home and/or public transportation.	7	6
Use of/availability of assistive technology (e.g., screen readers, amplification systems, screen magnifiers, and Bee Line reader).	7	6
Availability of a low sensory/low distraction place (e.g., quiet room with low overhead lighting/no fluorescent lights) for documentation or sensory breaks.	7	5
Extra time (e.g., for documentation, processing, treatment planning, and medication management).	6	6
Ability to modify policies of the fieldwork site to support students with disabilities (e.g., service animal in clinic, modified dress code, scent‐free products, ability to carry food/drink in the clinic, ability to carry smart device for medication reminders, and app use).	6	6
Dedicated/scheduled breaks in the student′s daily fieldwork schedule, for example, for lunch, using the bathroom and taking medication^a^.	6	6
Modified and/or flexible schedule for students.	6	5
Reduced productivity expectations for students.	6	5
Availability of physical assistance for transfers.	6	5
Slower increase in caseload^a^.	6	Missing

^a^New practice at Round 3, proposed from Round 2 comments. Reached consensus at Round 3.

## 4. Discussion

The results of this research provide a starting point for improving the accommodation of students with disabilities in OT clinical education. The experts′ recommendations share some similarities with recommendations of students in a recent qualitative study by Gregg et al. [51]. OT students with disabilities in Gregg et al.′s study stated that they appreciated faculty normalizing disability by proactively mentioning the possibility of requesting accommodations. They also appreciated universal accommodations (e.g., uploading lecture recordings for each class) that benefited all students, even without requesting formal accommodations. Students also highlighted the importance of supportive faculty attitudes and inclusive technical standards (baseline competency expectations). In light of their study findings, the authors call for inclusive technical standards, universal design for learning, normalizing disability accommodations in OT education, and affirming disability as a valuable form of diversity in OT [51].

One of the most striking observations for the research team, and something that experts also commented upon, was that many of these best practices would be useful both to students with and without disabilities. This is particularly the case for best practices under Theme 1: *facilitating communication*, *collaboration*, *and transparency*, including items about teaching and communication on fieldwork, including:

• FWE provides students with positive feedback (not exclusively critical feedback).
• FWE sets and communicates clear expectations for the student about the skills and progress they are meant to show on fieldwork.
• Regular check‐ins between student, site/FWE, and AFWC while on fieldwork to identify and address problems early (e.g., if student is not meeting expectations).
• AFWC provide templates and communication aids for conversations between FWE and student about accommodations, learning styles, and expectations.
• Regular check‐ins or feedback process for the student to provide feedback to the AFWC and the FWE about whether accommodations are effective and being provided as planned—without fear of retaliation.
• FWE provides student feedback in multiple formats (e.g., written, verbal, and video analysis).

Items from other themes are also appropriate for a universal approach for all students, including *FWEs teaching with the student′s learning preferences in mind* and *FWE creating an environment where the student feels safe to make mistakes*. These skills would be useful for clinical instructors to incorporate for all students. These best practices align well with principles for universal design in health profession clinical education created by Ireland′s Association for Higher Education, Access and Disability (AHEAD) and the School of Nursing at University College Dublin [52]. They conducted a positive enquiry workshop with 25 academics working in the health sciences who produced a list of nine principles for universal design for learning in clinical education. Several of these principles echo the findings of the present study, including the importance of clear communication about expectations, having a preplanned process for accommodations and advance planning including the benefits of preplacement site visits. For clinical educators, they recommend flexibility and a respect for “different ways of doing things” ([52], p. 476), communicating in a variety of ways, offering templates and resources for students, and cultivating recognition that mistakes are an important part of the learning process. They also recommend staff training to ensure confidence for working with disability accommodations, disability‐affirming attitudes, and defining required tasks for students “so they focus on what is to be done and not how, unless the how is a core skill” for example, wording a learning requirement about transfers as “ensure safe lifting of the patient rather than the student will lift the patient” ([52], p. 478). The best practices generated by this study and the principles outlined by Heelan et al. can serve as a baseline for clinical instructor education and self‐study to improve teaching skills. In addition, AFWCs, sites, and schools can utilize these best practices to better support clinical educators, for example, by providing templates to encourage conversations about learning styles and accommodations, or by setting a fixed schedule for regular check‐ins between the school, student, and FWE.

The present study′s findings also highlight the importance of changes that move beyond accommodating individual students in the current educational and clinical systems, towards structural and systemic change. The call for structural change in applied health professions has been made by disability studies scholars [43, 44, 53]. Individualized accommodations are essential, but they generally do not change the system for future students or practitioners. Structural changes such as rethinking productivity standards, designing workspaces with principals of universal design, and including disability studies or disability rights content in curriculum would have further reaching and longer term benefits for students and practitioners with and without disabilities. Eight best practices reached consensus for importance under the *Addressing Structural Barriers and Institutional Ableism* theme. Of these, six received a median feasibility score 5 or 6 with experts *somewhat agreeing* (5) or *agreeing* (6) that the items were feasible in the setting they were most familiar with. However, while items suggesting the profession rethink productivity standards received high rankings for importance, two of them (“reduced emphasis on productivity in the profession as a whole” and “Fieldwork educators have reduced productivity expectations when supporting any student (not just students with disabilities) during Level II fieldwork”) received median feasibility scores of *somewhat disagree* (3) or *neither agree nor disagree* (4), respectively. Changing the high productivity standards that American OTs currently work under would represent a massive systemic shift. Nonetheless, such a change is not impossible. Productivity standards based upon the ratio of direct patient contact time to documentation time do not exist in all settings or in all countries. Research demonstrates that higher productivity standards are associated with higher risks of practitioner burnout and unethical behaviors [54, 55] as well as higher risks of pressure injuries (pressure ulcers) and readmissions among patients due to reduced quality of patient care [56]. Changing approaches to practitioner productivity would not only make the profession more accessible to practitioners with disabilities but also could improve job satisfaction for nondisabled OTs and quality of care for patients. Of note, nine of the participants in the present study identified their primary setting as a skilled nursing facility, which have some of the highest productivity criteria among rehabilitation settings in the United States [55], with one study reporting an average productivity requirement of 85% [56]. In other settings and countries, standards about patient contact hours or billable hours can be much lower. In other environments, “productive” hours also sometimes include activities such as creating patient education materials, participating in training, seeking consults, or other necessary tasks, which are typically not included in US standards for “billable hours.”

One way to rethink the American model of productivity would be to take more of a team or collaborative approach to workload, rather than one focused on individual practitioner performance. For example, a rehabilitation unit could set goals as a group about how many patients they need to see, what training needs to be done, what splints or adaptive equipment needs to be made, and other tasks that need to be done. Then, these tasks could be collaboratively divided among the team based on their abilities. An OT practitioner who has educational expertise but needs to do fewer hours of direct patient care could be responsible for designing team in‐service trainings. Someone who is not particularly comfortable with group therapy could take more individual patients while leaving the groups to the colleague who excels in that setting. Some forms of collaborative work redistribution are already done in some units—even those with high productivity standards like where the third author previously worked—for example, when a team member was pregnant and needed to be off their feet more and lift less, other team members would switch some patients so this worker could have lighter or less dependent patients. This teamwork could be applied to disability accommodations as well. This study′s experts rated the item “Sites take a strong team/collaborative approach to workload and supervision rather than a competitive and productivity‐focused approach” with higher feasibility score than the other two productivity items, with a 5 (*somewhat agree*). Moreover, like the *reduced productivity expectations for FWEs*, it received the highest possible score for importance (7). The team approach could be a fruitful place to start making workplace changes.

Healthcare professional labor unions represent one avenue to advocate for the structural changes to workplace policies that affect people with disabilities. Labor unions can improve workplace conditions that represent social determinants of health for healthcare workers [57], thereby improving workers′ (including workers with disabilities) health. Some labor unions have also employed disability champions to specifically advocate for the rights of disabled employees [58]. In addition to positively affecting employee health outcomes, some research suggests that healthcare labor unions are associated with improved patient outcomes [59, 60]. Realizing the structural changes necessary to make the profession more accessible to disabled workers in the long term will require sustained, collective efforts—and labor unions have historically proven themselves to be one vehicle of such large‐scale workplace changes.

## 5. Limitations

Experts were all recruited from the United States to facilitate easy communication between experts about policies, terminology, and practices. While this somewhat limits the international generalization of the findings, in this article, we have endeavored to define and explain the US context and key actors to allow international readers to apply and adapt results to their local contexts. Additionally, although experts were purposively sampled based on published articles, group membership, and job roles, it is possible that the participants who were most passionate about the topic chose to participate, engendering a selection bias. This possibility was considered acceptable for the research, as the research indeed sought the expertise of those most experienced and motivated in the field, rather than an “average” practitioner, researcher, or student. Future research should apply these findings to a broader context, for example testing if training in these best practices is helpful to a generalist FWE who is not otherwise an expert in accommodations.

For the *suggested available accommodation* of “slower increase in caseload,” the Round 3 questionnaire did not collect feasibility ratings due to an error. As mentioned in the results section, in the Round 3 questionnaire, participants were reminded that importance scores were not meant to consider feasibility, because qualitative comments on Round 2 questionnaires indicated that participants were sometimes conflating importance and feasibility and rating less‐feasible items as less important. However, given that this reminder appeared only on the Round 3 survey, it is possible that the items that reached consensus for importance at Round 2 (and therefore did not appear on the final survey) were rated lower for importance compared with those at Round 3. Independent of this limitation, a difference in ratings at Rounds 2 and 3 is inherent to the multiround Delphi process, given that at Round 3 participants are also shown other experts′ ratings, in an effort to approach consensus. Delphi questionnaires were not pilot tested. As expected, some participant dropout occurred over the months of the study. The final sample remained above the preset minimum of 20 participants.

For the *suggested available accommodations*, our study used language to describe these accommodations that was compiled and edited from the comments of participants. The best practices written on the Delphi questionnaires did not use the type of precise phrasing that would need to appear on a formal accommodation letter. A participant who is a DRP approached the research team with this concern and suggested rewording, but as all rounds of the Delphi process had already been completed, the researchers retained the wording that had been agreed upon by participants. For suggestions of standardized language for formal accommodations, the authors suggest reviewing Dhanani et al.′s [61] article. The Job Accommodation Network (JAN) also has a helpful database of accommodations that is searchable by diagnosis, work function, topic, limitation, and accommodation [62]. Furthermore, Ozelie et al. [33] provide critical data on the perceived reasonableness of various accommodations across different clinical environments. Although the present study establishes a consensus on what practices are most supportive, Ozelie et al.′s work offers a practical lens through which AFWCs and students can anticipate potential institutional barriers or receptivity, thereby facilitating more collaborative and successful negotiation processes within specific OT settings.

## 6. Future Directions

Future research could test the implementation of these practices and examine how best practices in accommodations assist students with disabilities on fieldwork. Longitudinal studies that follow students through the fieldwork process would be of great interest, for example, with repeated interviews or surveys, or a daily diary methodology that asks students and/or educators for data at multiple intervals during fieldwork. Capacity building for FWEs and AFWCs is also an important direction for future practice and research, for example, through training and coaching in accommodation practices.

## 7. Conclusions

Improving the inclusion of practitioners with disabilities can play a role in heeding the longstanding calls to bring OT more into alignment with insights from disability studies and with the priorities of the disability community [43, 53, 63]. Better allying OT with disability communities and the fight for disability rights requires addressing “the frequent failure to consider disability in diversity and inclusion initiatives,” called out by OT and disability studies experts interviewed by Sheth et al. [53]. A diverse OT workforce has numerous benefits for students, practitioners, and the people OT practitioners serve [64]. Among these benefits is the potential for disabled practitioners to help address ableism in the profession. Practitioners′ experiences with ableism in OT shine a light on the ableism that must be addressed to better serve clients with disabilities [4, 65]. The inclusion of practitioners with disabilities can help healthcare professionals better address the health inequities facing people with disabilities [66].

Although it is essential to not lose sight of the broader structural changes that are needed to make the OT profession truly accessible and affirming to practitioners with disabilities, in presenting these results, the researchers have found that OT professionals are often eager to hear feasible recommendations they can start with *right now*. Ten of the best practices received a 7/7 median score for both importance and feasibility, and as such could offer a strong starting point. These practices are highlighted in Table 10.

**Table 10:** Top 10 most feasible practices.

Top 10 practices receiving 7/7 for importance and feasibility
Fieldwork educator sets and communicates clear expectations for the student about the skills and progress they are meant to show on fieldwork.
Fieldwork educator provides students with positive feedback (not only critical feedback).
Academic fieldwork coordinator should be knowledgeable about disability, accommodations, and disability policies.
Academic fieldwork coordinator advocates for and supports the student in accommodations process.
Transparency about the accommodations process—the school and fieldwork site keeping the student informed and included directly in conversations about accommodations.
Student engages in self‐advocacy for accommodations.
Academic fieldwork coordinator, faculty, and school′s disability services office educate students about accommodations early in the curriculum (e.g., first semester, with reminders each semester)
Fieldwork educator, academic fieldwork coordinator, and school′s disability services office support the student′s decision about whether or how to disclose disability and/or accommodation needs (e.g., help student weigh pros and cons of early disclosure, understand risks of disclosure, and respect student privacy).
Fieldwork educator, academic fieldwork coordinator, and school disability services office approach accommodations as an access and equity issue (a necessity and not a favor).
Fieldwork educator, academic fieldwork coordinator, and school′s disability services office conceptualize entry‐level practice expectations in a disability‐inclusive way (e.g., recognize that not all OT practitioners need to work full‐time and different settings have different demands).

In her seminal disability studies paper addressing OT and other “applied fields”, Simi Linton argued,

The influence and direction of disabled people should permeate the applied field. If rehabilitation professionals really believe in self‐determination for disabled people, they should practice what they teach by adhering to an active affirmative action program in their own departments and universities, adopting the books and essays of disabled people into their curricula, and by demanding that disabled people are in leadership positions in conference planning and on the platform at conferences, (1998, p. 531).

The authors join disability studies scholars and the expert participants in this study in calling for the OT profession to value disability as diversity, to affirm disability, and to actively recruit and support disabled students. We want our profession to not only comply with legal requirements for nondiscrimination but also create a culture that truly normalizes and celebrates disability.

## Author Contributions

**Courtney Gohean:** conceptualization, formal analysis, funding acquisition, funding acquisition, investigation, project administration, resources (lead), supervision, validation, visualization, writing – original draft, writing – review and editing. **Katie McLaughlin:** conceptualization, validation, visualization, writing – original draft, writing – review and editing. **Aster Harrison:** conceptualization, data curation, formal analysis (lead), funding acquisition, investigation, methodology (lead), project administration, resources, supervision, validation, visualization, writing – original draft, writing – review and editing.

## Funding

This study was supported by Faculty Development and Nexus Learning at Thomas Jefferson University.

## Conflicts of Interest

The authors declare no conflicts of interest.

## Endnotes

^1^There is ongoing discussion within the disability community regarding the use of person‐first language (e.g., “student with a disability”) versus identity‐first language (e.g., “disabled student”). This manuscript uses a combination of disability language to reflect the diversity of preferences within the community.

^2^In the United States, an entry‐level masters or doctoral degree is required to become an occupational therapist. The entry‐level degree to become an occupational therapy assistant (OTA) is an associate or bachelor′s degree. Both OTs and OTAs are then required to pass a national certification exam and apply for state licensure. Both OT and OTA students complete “Level I” (preliminary, shorter) clinical rotations as well as longer “Level II” rotations. Students are expected to reach entry‐level competence at the given site by the end of Level II placements.

## Supporting information


**Supporting Information** Additional supporting information can be found online in the Supporting Information section. Supporting Information. The three Delphi questionnaires are provided as supporting materials.

## Data Availability

Data are available upon request due to privacy/ethical restrictions.
