# Supplementary material for: Best Practices for Supporting Occupational Therapy Students With Disabilities During Clinical Education: Results From a Three‐Round Delphi Study
Source: Occup Ther Int. 2026 Sep 1;2026:6675487. doi: 10.1155/oti/6675487 (PMC13535157; doi:10.1155/oti/6675487)
Supplement: Supplementary file 1 [file OTI-2026-6675487-s001.docx]

Supplemental Materials: Best practices for supporting occupational therapy students with disabilities during clinical education: Results from a three-round Delphi study

**Delphi Questionnaires**

Delphi Round One Questionnaire

Instructions: In this survey, we will ask you for your opinions about fieldwork accommodations for students with disabilities on level II OT and/or OTA fieldwork. Please use the open-ended response boxes to list your responses. There is no word or character limit.

1. **In your opinion, what are the best practices in supporting and accommodating students with disabilities on level II fieldwork?**

You may want to consider some or all of the following questions: a. What actions can the site, the fieldwork educator, and/or the academic fieldwork coordinator do to support students with disabilities during fieldwork? b. What accommodations are helpful? c. What approaches to accommodations have been helpful? Please also feel welcome to share other ideas about best practices which are not captured by the prompts above.

2 **What features of the environment most contribute to the success of students with disabilities on level II fieldwork?**

You may consider any aspect of the social, physical or institutional environment, including: “the natural environment and human-made changes, support and relationships, products and technology, attitudes, and/or services, systems, and policies” (AOTA, 2020, pp. 36-39). You may want to consider: a. What social and attitudinal approaches are helpful? b. What forms of accessibility in the physical environment are helpful? c. What forms of assistive technology are helpful? d. What policies and systems best support students with disabilities?

3. **What challenges are associated with fieldwork accommodations for students with disabilities?**

These challenges could be associated with the environment, the profession, productivity demands, occupations, tasks, social relationships, student personal factors, etc.

4. **What would help address the challenges associated with fieldwork accommodations for students with disabilities?**

Here, think specifically about the challenges you named. What would help address these challenges?

Delphi Round Two Questionnaire

Instructions: In this survey, you will be shown a list of ideas for best practices in supporting students with disabilities while on Level II fieldwork. This list was developed by compiling the responses from the research participants (including you!) from the first Delphi survey. You will be asked to rate the **importance** and **feasibility** of each of the suggested best practices.

**Questions about Best Practices:**

**1.1 [Insert Best Practice]** *For example*: 1.1 Advance planning of accommodations with Fieldwork Educator and student well before fieldwork begins (involves early student disclosure of accommodation needs).

**Importance:** How much do you agree/disagree that the above practice is **important** for supporting students with disabilities on level II fieldwork?

- Strongly disagree (1)
- Disagree (2)
- Somewhat disagree (3)
- Neither agree nor disagree (4)
- Somewhat agree (5)
- Agree (6)
- Strongly agree (7)

**1.2 Feasibility:** How much do you agree/disagree that this practice is **feasible** for supporting students with disabilities on level II fieldwork? By feasible, we mean the extent to which the practice would be practical and possible to implement in the clinical setting you are most familiar with.

- Strongly disagree (1)
- Disagree (2)
- Somewhat disagree (3)
- Neither agree nor disagree (4)
- Somewhat agree (5)
- Agree (6)
- Strongly agree (7)

**1.3 Do you have any suggested edits to the phrasing or definition of this practice?**

**1.4** **Do you have any other comments about this practice?**

*This format (1.1 - 1.4) is repeated for each best practice.*

**Questions about Suggested Available Accommodations:**

*Intro text:* The final 12 items describe Level II fieldwork accommodations that participants’

suggested could be helpful for a variety of student needs. While accommodations

would be individualized and these accommodations would not be used for every

student, we are asking you to rate the importance and feasibility of these

accommodations being available to students who need them.

**2.1 [Insert Suggested Available Accommodation]** *For example:* 2.1 Modified and/or flexible schedule

**2.1 Importance:** How much do you agree/disagree that the above practice is **important** for supporting students with disabilities on level II fieldwork?

- Strongly disagree (1)
- Disagree (2)
- Somewhat disagree (3)
- Neither agree nor disagree (4)
- Somewhat agree (5)
- Agree (6)
- Strongly agree (7)

**2.2 Feasibility:** How much do you agree/disagree that this practice is **feasible** for supporting students with disabilities on level II fieldwork? By feasible, we mean the extent to which the practice would be practical and possible to implement in the clinical setting you are most familiar with.

- Strongly disagree (1)
- Disagree (2)
- Somewhat disagree (3)
- Neither agree nor disagree (4)
- Somewhat agree (5)
- Agree (6)
- Strongly agree (7)

**2.3 Do you have any suggested edits to the phrasing or definition of this practice?** (Open response)

**2.4 Do you have any other comments about this practice?** (Open response)

*This format (2.1 - 2.4) is repeated for each suggested available accommodation.*

**3.0 Final Survey Questions:**

3.1 **After reading this list of best practices, do you have any other ideas for best practices that are not included on this list?** (Open response)

3.2 **Do you have any other comments or ideas you’d like to share?** (Open response)

Delphi Round Three Questionnaire

Instructions: In this survey, you will be shown a list of 6 ideas for practices for better supporting OT/OTA students with disabilities on Level II fieldwork. This list was developed by compiling the practices from round 2 of this Delphi study that did not reach consensus for importance (were not agreed upon) in Round 2. In Round 2, you rated each item on a 7-point scale for importance (where 1 meant that you "strongly disagree" that the practice is/would be important, and 7 meant that you "strongly agree" that the practice is/would be important).  In this final survey, you will be shown these remaining practices and the median importance score that other participants gave each practice. You will be given the opportunity to change the rating that you gave the practice at Round 2 upon consideration of others’ score. Please note that while you are able to adjust your score if you choose, you are **not required** to change your score. In addition, you will be shown 2 new ideas for best practices that were proposed by participants in the comments during Round 2. You will be asked to give these items a score for importance and feasibility.

**Questions about Best Practices:**

**1.1 [Insert Original Best Practice]** *For example:* "Standardized accommodation process at school and sites (consistent process/timeline across students, even if individual accommodations are tailored to each student)."

**Suggested new wording based on feedback:**

**"**Schools and sites have a developed, standard, step-by-step process for how they implement accommodations. For example, they would have a readily-available list of the steps of the process, accommodations commonly used, key people to involve, and other information necessary to the process. Each school and site's process could be different, but within their organization they would have a "standard of practice" or workflow/checklist established."

**1.1 Change Decision:**

You rated this item as [insert numerical and descriptive rating from the participant at round 2, for example “4, neither agree nor disagree"]. Other participants gave this item a median rating of "Agree" (6 out of 7). **Please remember that when you are scoring items for importance, you do not need to consider feasibility**. Would you like to change your importance rating?

- Yes
- No

**1.2 Rating Change (*if yes to previous question)*:**

After considering the median response shown on the previous screen, how much do you agree/disagree that this practice is important for supporting students with disabilities on level II fieldwork?

- Strongly disagree (1)
- Disagree (2)
- Somewhat disagree (3)
- Neither agree nor disagree (4)
- Somewhat agree (5)
- Agree (6)
- Strongly agree (7)

**1.3 Do you have any other comments about this practice?** For example, you could suggest additional rewording or write here if you want to add clarity about why or why not the practice is important and/or feasible in your clinical setting.

*This format (1.1-1.3) is repeated for each practice.*

**Questions about Suggested Available Accommodations:**

**2.1 [Insert proposed accommodation]** *For example****:***

*“***Original Best Practice**: "Reduced caseload or a slower increase in caseload."

*This practice was suggested in round 1 as a Level II fieldwork accommodation that participants’ suggested could be helpful for a variety of student needs. While accommodations would be individualized and these accommodations would not be used for every disabled student, we are asking you to rate the importance of this accommodation being available to students who need it.

**Suggested new wording based on feedback:** "Slower increase in caseload"

(We have separated "reduced caseload" and "slower increase in caseload" into 2 separate items. Please rate "slower increase in caseload" here.)

**2.1 Change Decision:**

You rated this item as [insert numerical and descriptive rating from the participant at round 2, for example “4, neither agree nor disagree"]. Other participants gave this item a median rating of "Agree" (6 out of 7). **Please remember that when you are scoring items for importance, you do not need to consider feasibility**. Would you like to change your importance rating?

- Yes
- No

**2.2 Rating Change (*if yes to previous question)*:**

After considering the median response shown on the previous screen, how much do you agree/disagree that this practice is important for supporting students with disabilities on level II fieldwork?

- Strongly disagree (1)
- Disagree (2)
- Somewhat disagree (3)
- Neither agree nor disagree (4)
- Somewhat agree (5)
- Agree (6)
- Strongly agree (7)

**2.3 Do you have any other comments about this practice?** For example, you could suggest additional rewording or write here if you want to add clarity about why or why not the practice is important and/or feasible in your clinical setting.

*This format (2.1-2.3) is repeated for each suggested available accommodation.*

**Questions about New Items not Present at Round Two:**

***Intro text:*** The next two practices were not included in the previous round of rankings. These are ideas for new practices that were proposed by participants during the Round 2 survey.

**3.1 [Insert proposed accommodation]** *For example****:*** Proposed new practice #1: Training for university Student Disability Services staff (Disability Resource Professionals/DRPs) providing background about OT/OTA, clinical rotations/fieldwork and clinical accommodations to better prepare DRPs to support OT/OTA students with disabilities.

How much do you agree/disagree that this practice is/would be **important** for supporting students with disabilities on level II fieldwork?

- Strongly disagree (1)
- Disagree (2)
- Somewhat disagree (3)
- Neither agree nor disagree (4)
- Somewhat agree (5)
- Agree (6)
- Strongly agree (7)

**3.2** How much do you agree/disagree that this practice is/would be **feasible** for supporting students with disabilities on level II fieldwork? By feasible, we mean the extent to which the practice would be practical and possible to implement in the clinical setting you are most familiar with.

- Strongly disagree (1)
- Disagree (2)
- Somewhat disagree (3)
- Neither agree nor disagree (4)
- Somewhat agree (5)
- Agree (6)
- Strongly agree (7)

**3.3** Do you have any suggested edits to the phrasing or definition of this practice?

**3.4** Do you have any other comments about this practice? For example, you could suggest additional rewording or write here if you want to add clarity about why or why not the practice is important and/or feasible in your clinical setting.

*This format (3.1-3.4) is repeated for each new practice.*

**Final Question:**

**4.0 Do you have any other comments or ideas you would like to share?**
